# Phenolic Compounds Profile and Antioxidant Capacity of Plant-Based Protein Supplements

**DOI:** 10.3390/molecules29092101

**Published:** 2024-05-02

**Authors:** Tomasz Sawicki, Monika Jabłońska, Anna Danielewicz, Katarzyna E. Przybyłowicz

**Affiliations:** 1 Department of Human Nutrition, The Faculty of Food Science, University of Warmia and Mazury in Olsztyn, Słoneczna 45F, 10-718 Olsztyn, Poland; monika.jablonska@uwm.edu.pl (M.J.); anna.danielewicz@uwm.edu.pl (A.D.); katarzyna.przybylowicz@uwm.edu.pl (K.E.P.); 2 College of Medical Sciences in Olsztyn, Nicolaus Copernicus Superior School, Nowogrodzka 47A, 00-695 Warsaw, Poland

**Keywords:** flavonoids, phenolic acids, plant products, proteins, antioxidant activity

## Abstract

The study aimed to determine the phenolic content and antioxidant capacity of five protein supplements of plant origin. The content and profile of phenolics were determined using the UHPLC-DAD-MS method, while antioxidant capacity (ABTS and DPPH assays) and total phenolic content (TPC) were evaluated using spectrophotometric tests. In the analyzed proteins, twenty-five polyphenols were detected, including eleven phenolic acids, thirteen flavonoids, and one ellagitannin. Hemp protein revealed the highest individual phenolics content and TPC value (1620 μg/g and 1.79 mg GAE/g, respectively). Also, hemp protein showed the highest antioxidant activity determined via ABTS (9.37 μmol TE/g) and DPPH (9.01 μmol TE/g) assays. The contents of *p*-coumaric acid, *m*-coumaric acid, kaempferol, rutin, isorhamnetin-3-*O*-rutinoside, kaempferol-3-*O*-rutinoside, and TPC value were significantly correlated with antioxidant activity assays. Our findings indicate that plant-based protein supplements are a valuable source of phenols and can also be used in research related to precision medicine, nutrigenetics, and nutrigenomics. This will benefit future health promotion and personalized nutrition in the prevention of chronic diseases.

## 1. Introduction

Protein supplements have become a common part of modern diets, offering a practical and effective way for individuals to meet their daily protein requirements. This aspect is critically essential for populations such as athletes, people engaged in physically demanding jobs, and individuals with specific dietary preferences who rely on protein supplements to aid in muscle development, enhance post-workout recovery, and support overall health [1]. Protein supplements have been derived from both animal and plant sources. However, a noticeable shift towards plant-based protein supplements is currently occurring [2]. An increasing awareness of dietary choices, environmental concerns, and general health implications drives this trend. Plant-based options are favored for their ability to meet daily protein requirements and their alignment with vegetarian, vegan, and planetary health diets. These dietary approaches prioritize reducing the consumption of animal products and, as a consequence, improving human health and reducing environmental impacts [3,4].

Currently, a plethora of plant-based supplements can be found on the market. Some of the most popular protein supplements are those made from peas, rice, soy, hemp, and pumpkin seeds. Pea protein provides essential amino acids crucial for muscle growth and repair and contains plenty of fiber, supporting digestive health [5]. Rice proteins, characterized by their hypoallergenic properties, make a great alternative for people with sensitivities to dairy or soy [6]. Soy protein, in turn, has been extensively studied for its potential to lower cholesterol and reduce the risk of heart disease [7]. Hemp protein is rich in omega-3 and omega-6 fatty acids and contributes, among others, to cardiovascular health [8]. Pumpkin seed protein offers a rich array of minerals, including magnesium and zinc, which are essential for various metabolic processes and immune functions [9]. Each of these protein sources offers distinct nutritional benefits. Therefore, there is a need for further investigation into their holistic health implications.

It is widely known that plants serve as rich sources of phytonutrients, among them phenols, which are not present in animal-based products. Phenols are a diverse group of phytochemicals, encompassing a wide range of compounds, including flavonoids and phenolic acids. They are abundant in fruits, vegetables, nuts, seeds, and beverages (e.g., tea and red wine) [10]; however, to the best of our knowledge, their concentration and bioavailability in protein supplements remain underexplored. Phenols are known for their antioxidant properties, contributing to reducing oxidative stress and inflammation [11]. These substances also modulate the gut microbiota, enhancing nutrient absorption and potentially reducing the risk of various lifestyle-related diseases. Incorporating polyphenol-rich foods into one’s diet is often recommended as part of a healthy lifestyle [12,13,14].

In light of the recognized nutritional advantages of plant-based protein supplements and the underexplored domain of their phenolic content, the current study aimed to investigate the presence of phenols in protein supplements derived from pea, rice, soy, hemp, and pumpkin seeds. We applied High-Performance Liquid Chromatography–Diode Array Detector–Mass Spectrometry (HPLC-DAD-MS) to meet this goal. By elucidating the presence and concentration of polyphenols in plant-based protein supplements, we seek to provide practical insights for consumers who want to make informed dietary decisions and for manufacturers interested in improving the nutritional value of their products. The results of this study endeavor to shed light on the holistic health implications of plant-based protein supplements, offering a foundation for future research and innovation in the field of nutritional science.

The conducted research can also be used in nutritional epigenetics, examining changes in gene expression induced by bioactive dietary ingredients, the profile of which should be determined. This new approach in scientific work is encouraging, as it will provide new knowledge in the context and era of precision medicine, nutrigenetic and nutrigenomic research offers significant opportunities to improve the prevention of metabolic disorders such as cancer, diabetes, hypertension, and cardiovascular diseases [15].

## 2. Results and Discussion

### 2.1. Phenolic Content and Composition

To the best of our knowledge, this is the first study to demonstrate the presence of phenolic compounds in commercially available plant-based protein supplements. While the majority of studies have focused on the presence of phenols in raw plant material [16] and their derived products [17] or as additives to increase the bioactive properties of protein supplements [18], there has been a notable gap in the investigation of these bioactive compounds in the context of plant-based protein supplements. Therefore, this study uniquely presents the profile and content of phenolic compounds and antioxidant capacity of five different plant-based protein supplements (soy, rice, pea, hemp, and pumpkin seed proteins), with the aim of broadening our understanding of their nutritional value beyond their protein contribution.

In the current study, twenty-five polyphenols were detected and quantified in plant-based protein supplements (Table 1) using the UHPLC-DAD-MS method. Eleven of the phenols were phenolic acids (gallic, *p*-coumaric, gentistic, caffeic, syringic, vanillic, benzoic, m-coumaric, salicylic, ferulic, and o-coumaric acids), thirteen compounds belonged to flavonoids ((+)-catechin, isorhamnetn-3-*O*-glucosied, kaempferol, myricetin, quercetin-3-*O*-glucoside, rutin, isorhamnetn-3-*O*-rutinoside, quercetin-3-*O*-galactoside, quercetin-3-*O*-vicianoside, kaempferol-3-*O*-rutinoside, quercetin, naringenin, and apigenin), and one represented ellagitannins (ellagic acid). Among the identified phenolic acids, five belonged to hydroxycinnamic acids (caffeic, *p*-coumaric, *m*-coumaric, *o*-coumaric, and ferulic acids), while six compounds belonged to hydroxybenzoic acids (gallic, gentisic, syringic, vanillic, benzoic, and salicylic acids). Gallic acid, *p*-coumaric acid, gentisic acid, vanillic acid, benzoic acid, salicylic acid, ferulic acid, and quercetin were found in all examined protein samples. Interestingly, benzoic acid was found to be the predominant compound in protein isolates obtained from rice, pea, hemp, and pumpkin seeds. Benzoic acid is known for its antimicrobial properties, effectively inhibiting the growth of bacteria and yeast. Consequently, it is commonly used as a preservative to extend the shelf life of various food products [19]. While generally recognized as safe for consumption in limited amounts, the excessive intake of benzoic acid could potentially lead to health issues, such as allergic reactions [20]. The significant concentration of benzoic acid detected in plant-based protein supplements raises intriguing questions. One possibility is that benzoic acid may result from the dehydroxylation of *p*-hydroxybenzoic acid during protein isolate production under certain conditions (e.g., elevated temperatures or specific catalytic reactions). This hypothesis is supported by the previous literature reports from other researchers who have identified *p*-hydroxybenzoic acid in the raw material of these supplements [16,21,22,23]. On the other hand, benzoic acid could be intentionally added as a preservative in the examined protein isolates. However, none of the tested plant-based protein supplements included information about the presence of benzoic acid on their packaging labels despite its significant concentration in the products.

The highest number of phenolic compounds were identified in soy protein supplements (19 compounds), including 9 compounds of phenolic acids and flavonoids each and 1 ellagitannin. The most abundant polyphenol in this sample was ellagic acid, with a 19.7% contribution to the total phenolic index (TPI). Moreover, the ellagic acid content found in the current study for this sample was more than two and four times higher than rice and pumpkin seed proteins, respectively (Table 1). The significant contribution of gentisic acid and benzoic acid also characterized the soy protein.

It should be emphasized that soy protein isolate was characterized by more detected flavonoids than the other analyzed plant protein samples. The flavonoid profile of soy protein includes (+)-catechin, kaempferol and its derivatives, myricetin, rutin, quercetin, naringenin, and isorhamnetin derivatives. The concentration of (+)-catechin was more than three and seven times higher than pea and pumpkin seed protein samples, respectively. Flavonoids identified in soy-based protein isolates are known to improve cardiovascular health by enhancing blood circulation, reducing inflammation, and offering lipid-lowering benefits crucial for metabolic syndrome prevention [24]. The enriched flavonoid composition of soy protein highlights its potential as a multifunctional food source, offering broader health benefits than other protein supplements.

The following protein sample with a high number of detected phenols (18) was a pumpkin seed protein supplement. It contained nine phenolic acids, eight flavonoids, and ellagic acid (Table 1). The most abundant compound in pumpkin seed protein was benzoic acid (37.8% of the TPI), followed by gentisic acid (26.7% of the TPI) and salicylic acid (10.2% of the TPI). The contribution of the other identified phenols in pumpkin seed protein samples was below 5% of the TPI. Interestingly, two detected compounds, i.e., gentisic and salicylic acids, were found in significantly (*p* < 0.05) higher concentrations than the other analyzed samples. The pumpkin seed sample showed an average 94% higher concentration of gentisic acid than other analyzed protein samples and more than 90% higher salicylic acid content than soy, rice, and pea proteins. Both gentisic and salicylic acids possess notable antioxidant and anti-inflammatory properties. Gentisic acid is thought to promote cardiovascular well-being by modulating cholesterol levels and protecting against atherosclerosis [25]. Salicylic acid, in turn, is recognized for its wide-ranging benefits, including anti-inflammatory, anticancer, neuroprotective, and antidiabetic effects [26]. Given its rich content of both gentisic and salicylic acids, pumpkin seed protein offers a unique spectrum of protective benefits.

In the hemp protein supplement, we identified 17 phenols, consisting of 10 phenolic acids and 7 flavonoids, representing the highest phenolic acid count compared to all other examined samples. Similar to rice, pea, and pumpkin seed protein samples, hemp protein samples were dominated by benzoic acid. Rutin, salicylic acid, and kaempferol-3-*O*-rutinoside contributed significantly to the TPI (10.3%, 8.9%, and 7.2%, respectively), while the other identified phenols in hemp protein isolate collectively contributed less than 5% to TPI. Among the analyzed samples, hemp protein was characterized by the highest content of two phenolic acids (gallic and caffeic acids) and four flavonoids (kaempferol, rutin, isorhamnetin-3-*O*-rutinoside, and kaempferol-3-*O*-rutinoside). The most significant differences in concentration among the samples were observed for rutin. This flavonoid was found only in two samples (hemp and soy proteins), with the twenty-sixth highest concentration in hemp protein. Hemp protein was also a good source of kaempferol-3-*O*-rutinoside with more than two-, three-, and five-fold higher content than soy, rice, and pumpkin seed proteins, respectively. Additionally, hemp protein contained approximately twice the amount of gallic acid content compared to the other analyzed protein samples. The remarkable concentration of rutin and kaempferol-3-*O*-rutinoside in hemp protein enhances its value as a superior nutritional supplement with antioxidative and anti-inflammatory properties crucial for preventing chronic diseases and supporting overall health [27].

Pea-based protein supplements contained 15 phenolic compounds, including 9 phenolic acids and 6 flavonoids. Like the other analyzed samples, benzoic acid was the predominant compound in pea protein (59.3%). Syringic acid (6.6%), quercetin-3-*O*-vicianoside (8.0%), and quercetin (6.5%) made significant contributions to the TPI in pea protein. Other identified phenols contributed less than 5% to the TPI. It should be noted that syringic acid was exclusively identified in pea protein, distinguishing it from the other examined samples. This phenolic acid demonstrates antioxidant, anti-inflammatory, antimicrobial, and neuroprotective properties, contributing to the prevention of various conditions, including diabetes, cardiovascular diseases, cancer, and cerebral ischemia [28,29]. Pea protein was also characterized by the highest concentrations of quercetin glycosides (quercetin-3-*O*-glucoside, quercetin-3-*O*-galactoside, and quercetin-3-*O*-vicianoside) and apigenin. Among these, quercetin-3-*O*-vicianoside content was significantly higher in pea protein compared to pumpkin seed and hemp protein samples. Apigenin levels were 35% higher in pea protein than in rice protein. The results of the studies suggest that apigenin may reduce the secretion of key proinflammatory cytokines, including TNF-α, IL-1β, IL-6, and IL-10, and, therefore, shows promise for managing various inflammatory conditions like cancer, diabetes, cardiovascular, and neurodegenerative diseases [30].

The rice protein supplement had the lowest number of phenols (13 phenols), including 8 phenolic acids, 4 flavonoids, and 1 ellagitannin. As observed in the other samples, benzoic acid dominated, with the highest contribution among all analyzed protein samples, constituting 70.1% of the TPI (Table 1). The second most prominent compound observed in rice protein was quercetin, constituting 7.0% of the TPI. Other detected phenols comprised less than 5% of the TPI. Rice protein was characterized by a significantly higher ferulic acid and naringenin content compared to the other plant-based protein samples. Ferulic acid concentration was 36.43 µg/g and was approximately two times higher than the other analyzed protein plant-based supplements. The concentration of naringenin in rice protein was more than 10% higher than in soy and pumpkin seed protein samples. Ferulic acid presents notable therapeutic potential in addressing conditions like diabetes, cancer, and cardiovascular diseases, primarily due to its antioxidant and anti-inflammatory properties. It exhibits various biological activities including anticarcinogenic, antiallergic, antimicrobial, and hepatoprotective effects, making it a valuable compound for medical research and clinical applications [31]. Naringenin, in turn, exhibits a wide range of potential health benefits, including antioxidant, anti-inflammatory, antidiabetic, anti-hypertensive, neuroprotective, and cardioprotective properties. Although most of the evidence comes from in vitro and animal studies, clinical trials focusing on cardioprotective effects have shown promising results, particularly in patients with cardiovascular risk factors [32].

As mentioned above, to the best of our knowledge, there is no information on the profile and content of phenols in the analyzed protein plant-based supplements. Therefore, our results were compared with available data on phenols identified in row material (soybeans, rice, peas, hemp, and pumpkin seeds) or derived products. In the case of soybeans, the results of the study by Zhu et al. [23] pointed to the presence of 8 phenolic acids in 18 soybean cultivars. In the above research, the soybeans did not contain flavonoids, ellagitannins, and six phenolic acids (gentisic, vanillic, benzoic, *m*-coumaric, salicylic, and *o*-coumaric acids) identified in our study. On the other hand, four phenolic acids (protocatechuic, chlorogenic, *p*-hydroxybenzoic, and cinnamic acids), which were detected in soybeans from China, were not found in soy proteins [23]. The same authors showed that the primary phenolic acids in the analyzed soybeans were chlorogenic, *p*-hydroxybenzoic, and caffeic acids in the black soybean samples. In contrast, protocatechuic and *p*-coumaric acids were the major compounds in yellow soybean cultivars [23]. In comparison, 14 different soybeans also from China examined by Wang et al. [33] were characterized by the presents of 6 phenolic acids (gallic, *p*-coumaric, syringic, vanillic, ferulic, and protocatechuic acids) and 4 flavonoids (rutin, quercetin, epicatechin, and isoquercetin). The cited study did not detect the five phenolic acids and eleven flavonoids that were noted in our study. Ma et al. [34] analyzed nine brown and white japonica rice cultivars; results showed the presence of only six phenolic acids (caffeic, sinapic, ferulic, *p*-hydroxybenzoic, syringic, and *p*-coumaric acids). Moreover, as in soybeans, *p*-hydroxybenzoic acid was the predominant phenolic acid in the analyzed japonica rice. Results of a study by Li et al. [22] showed the presence of eleven phenolic acids in seven varieties of brown rice from southern China. In the above study, the rice samples did not contain the six phenolic acids (gallic, gentistic, benzoic, m-coumaric, salicylic, and *o*-coumaric), flavonoids, and ellagitannins that were detected in our study. On the other hand, four phenolic acids (protocatechuic, *p*-hydroxybenzoic, chlorogenic, and *trans*-3-hydroxycinnamic acids), which were found in rice samples [22], were not detected in rice protein isolate. Pumpkin seeds and their roasted products were characterized by the presence of seven phenolic compounds, including five phenolic acids (gallic, caffeic, *p*-hydroxybenzoic, ferulic, and *p*-coumaric acids) and two flavonoids (epicatechin and rutin) [17]. The results presented by Peng et al. [17] showed that *p*-hydroxybenzoic acid had the largest share in the content of polyphenolic compounds in the analyzed pumpkin seeds and pumpkin seed products. In turn, Foss et al. [16] showed the presence of 26 phenolic compounds in Indian hemp, including 12 phenolic acids, 8 flavonoids, and 7 stilbenes. In the cited study, the predominant compound was orientin, with approximately 25% contribution of TPI; moreover, this flavonoid was not detected in our samples. In comparison, Izzo et al. [35] detected twenty-two polyphenols in hemp from four different subclasses (phenolic acids, ligninamides, phenolic amides, and flavonoids) of these compounds. In the case of the pea, the available literature shows that a total of 115 structurally different phenolic compounds are present in peas, most of which are glycosylated flavonols (mainly 3-*O*-glycosides of kaempferol and quercetin) along with their biosynthetically related counterparts [21].

The total phenolic index (TPI) was found within 601.34–1620.69 µg/g (Table 1). The highest value of TPI was determined in the hemp protein sample, while the lowest value was pointed in soy protein. No statistical differences (*p* > 0.05) in the TPI values were found between the hemp and pumpkin seed proteins, as well as between rice and pea protein samples. The highest TPI values in hemp and pumpkin seed proteins can be attributed to the fact that these samples were characterized by the highest number of detected polyphenols (17 and 18 compounds, respectively). Hemp and pumpkin seed protein samples also showed more than 90% higher concentrations of salicylic acid than the soy, pea, and rice protein supplements. Moreover, as mentioned above, hemp protein demonstrated the highest content of rutin and kaempferol-3-*O*-rutinoside, while pumpkin seed protein was characterized by the highest concentration of gentisic acid (Table 1).

**Figure 1:**
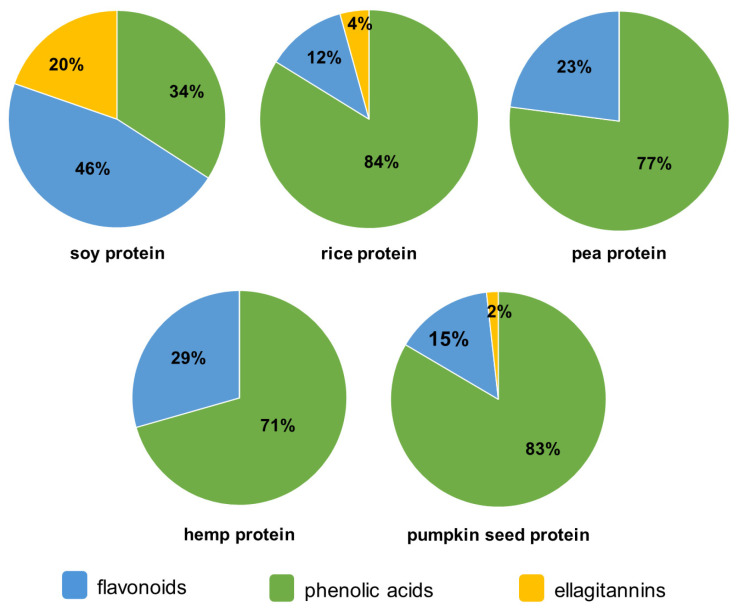
Pie charts showing the average content of phenolic acids, flavonoids, and ellagitannins in plant-based protein supplements.

The dominant group of phenolic compounds in four out of five tested plant-based protein supplements were phenolic acids (Figure 1). Phenolic acids were dominated in rice, pea, hemp, and pumpkin seed protein supplements with an average contribution of 79% of the TPI. On the other hand, flavonoids were the leading group in the soy protein (46%), while phenolic acids constituted 12% less of the TPI. As mentioned above, ellagitannins were also found in examined samples (Table 1), which were represented by one compound (ellagic acid). This group was detected in three protein samples (soy, rice, and pumpkin seed proteins), with the highest contribution of ellagitannins found in soy protein (20%). Ellagic acid is recognized for its wide range of health benefits. It has been extensively studied for its antioxidant properties, attributed to its ability to scavenge free radicals and inhibit oxidative stress-induced damage to cells and tissues. It also exhibits anti-inflammatory, anti-carcinogenic, and anti-mutagenic effects, underscoring its potential as a multifaceted therapeutic agent. The results of the studies also suggest that ellagic acid supports gastrointestinal health [36,37]. The presence of ellagic acid in soy, rice, and pumpkin seed protein isolates further supports their health-promoting potential.

### 2.2. Total Phenolic Content (TPC), Antioxidant Capacity, and Correlation Study

**Figure 2:**
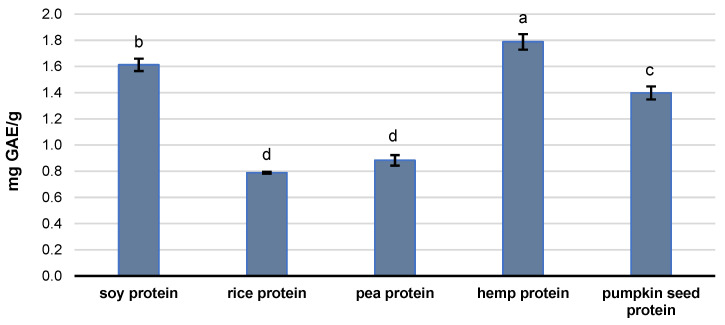
Total phenolic content (TPC) determined in plant-based protein supplements. Data are a means ± standard deviation (n = 3). Different letters indicate statistical significance (*p* ≤ 0.05).

The results presented in Figure 2 demonstrate the total phenolic content (TPC) in the tested plant-based protein supplements. As shown, hemp protein was characterized by a TPC value more than twice that of rice and pea protein supplements. Similarly, the TPC value for soy proteins was approximately twice as high as for rice and pea protein samples. Moreover, hemp protein supplements showed 22% higher TPC content than pumpkin seed protein. Pumpkin seed protein was characterized by 37 and 43% higher TPC contents than rice and pea protein supplements, respectively.

As shown in Figure 2, the TPC in pumpkin seed protein isolate was 1.40 mg of GAE/g, while the data presented by Nkosi et al. [38] indicated that this protein sample had a higher TPC value of 2.30 mg of GAE/g. The TPC values in 30 soybean samples ranged from 2.07 to 9.01 mg of GAE/g, while black soybeans were characterized by higher phenolic content than yellow soybeans [39]. In our previous study, Indian hemp was characterized by a level of free TPC at approximately 2 mg of GAE/g. Moreover, our data showed that hemp’s phenolic compounds are primarily present in bound forms [16]. Also, a similar observation was reported by Li et al. [22], which showed that the contribution of the bound phenolic compounds from seven rice varieties accounted for 6.25–41.86% of the TPC in the obtained extract. This information can explain the relatively high content of phenols in plant-based proteins. The available literature shows phenolic acids can form ester linkages with structural carbohydrates and proteins through their carboxylic group [40]. During the production of plant protein supplements, thermal denaturation and leaching with water or aqueous ethyl alcohol solution are used [41], which may lead to the release of polyphenolic compounds from ester and glycosidic bonds.

The antioxidant capacities of plant-based protein supplements were examined using ABTS and DPPH assays. Among the analyzed protein isolates, hemp protein exhibited the highest antioxidant activity, with ABTS and DPPH values of 9.37 TE/g and 9.01 TE/g, respectively (Table 2). The obtained results are consistent with those from previous studies, where hemp protein isolate exhibited comparable antioxidant activity levels [42]. Pumpkin seed and soy proteins also demonstrated notable antioxidant activities, indicating their potential to combat oxidative stress. In contrast, rice protein demonstrated the lowest antioxidant potential. Pea protein exhibited a lack of DPPH activity and relatively lower ABTS activity.

Thanks to their potent antioxidant properties, polyphenols present in plant-based protein supplements may offer numerous benefits to physically active individuals. During exercises, elevated oxygen consumption in the body leads to the increased production of free radicals, which may damage muscle tissue and impede muscle regeneration and growth. Various studies suggest that polyphenols play a key role in maintaining muscle mass and functionality by inhibiting proatrophic factors and signaling pathways that are important in the degradation of muscle proteins. In addition, polyphenols may promote muscle protein synthesis and myogenesis and improve the quality and function of mitochondria [43,44,45]. Furthermore, it was also demonstrated that polyphenols can modulate the immune system through interactions with the intestinal microflora. By influencing the production of metabolites such as butyrate, which regulates cytokine production and maintains the integrity of the intestinal barrier, polyphenols may regulate the pro- and/or anti-inflammatory balance crucial for post-exercise recovery [46]. Antioxidants present in plant-based protein supplements may potentially improve the efficiency of protein digestion by protecting digestive enzymes from oxidative stress [47]. In consequence, this can lead to a more efficient protein breakdown, absorption, and utilization of amino acids, which, in turn, are necessary for muscle repair and growth.

It should be emphasized, however, that the antioxidant activities observed in the examined protein isolates might not solely derive from their phenolic content. It was demonstrated that proteins themselves, due to their amino acid composition and the presence of specific peptides, may also interact with free radicals [48]. Further studies focusing on protein profiles could provide more insight into their particular roles in contributing to the antioxidant capacity of plant-based protein supplements.

**Figure 3:**
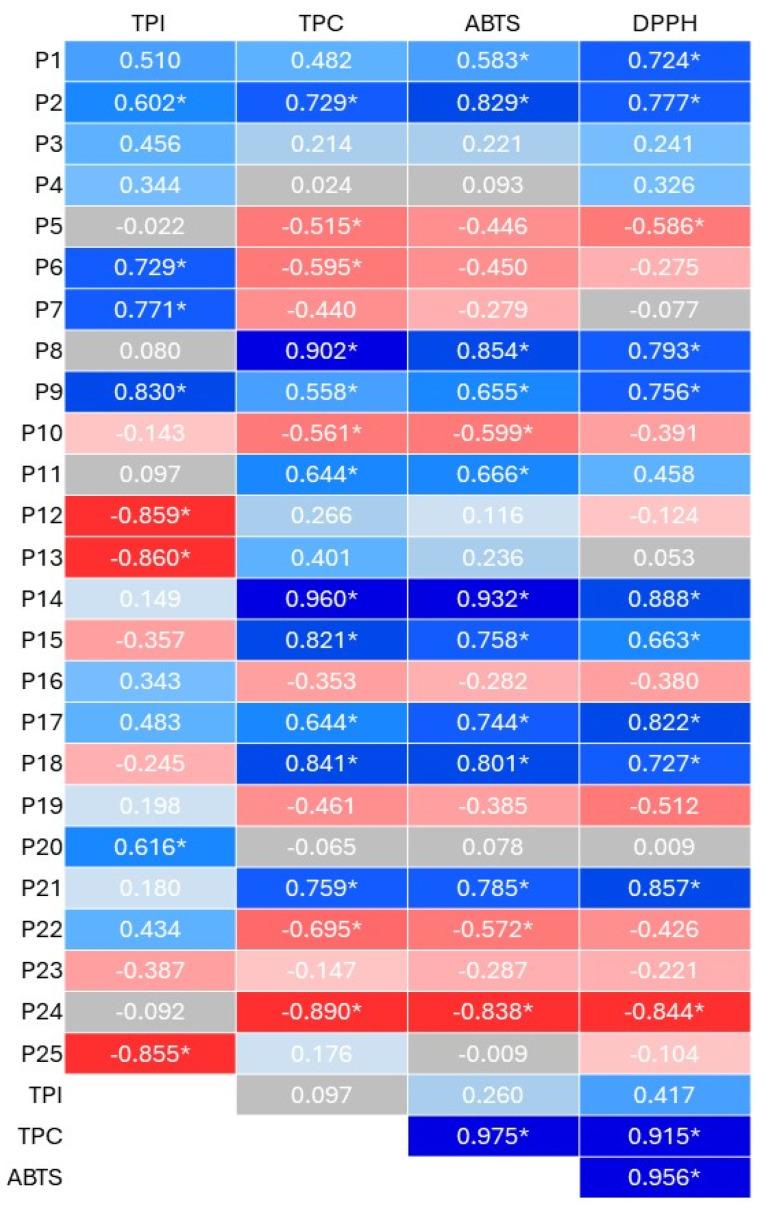
Pearson’s correlation coefficients (r) heat map of antioxidant properties (ABTS, DPPH; µmol TE/g), total phenolic content (TPC) and index (TPI), and the content of individual phenolic compounds (identified in plant-based protein supplements; µg/g) (P1–P25 represent the number of polyphenols identified in plant-based protein supplements—Table 1); * *p* < 0.05.

A correlation analysis revealed a significant positive link between ABTS, DPPH values, and the concentrations of kaempferol, kaempferol-3-*O*-rutinoside, and m-coumaric acid within these supplements, emphasizing their robust antioxidant profiles (Figure 3). The absence of these compounds in rice and pumpkin seed proteins may explain their comparatively lower antioxidant activity. This finding aligns with prior research confirming the potent antioxidant properties of kaempferol [49]. Moreover, the heat map also showed that the ABTS and DPPH assays were highly positively correlated with the content of *p*-coumaric acid, rutin, isorhamnetin-3-*O*-rutinoside, and TPC value. In addition, the highest antioxidant capacity of hemp protein may be due to the presence of these mentioned components and the combined/synergistic effect of kaempferol, kaempferol-3-*O*-rutinoside, and m-coumaric acid.

**Figure 4:**
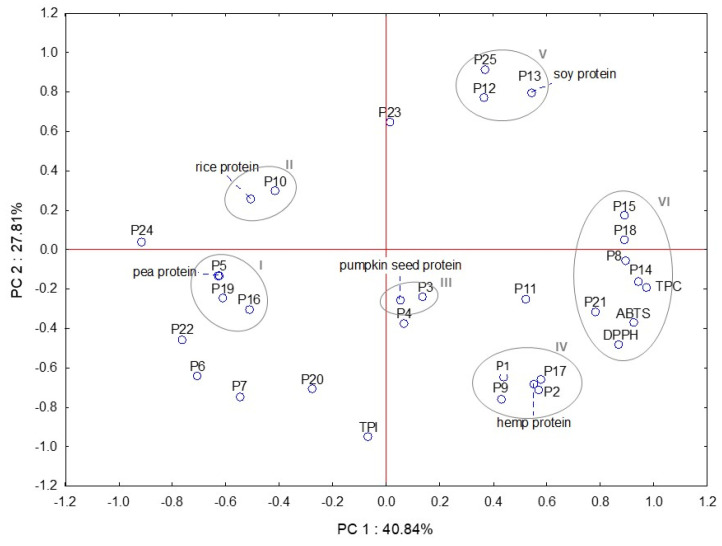
Principal component scatterplot of plant-based protein supplements, antioxidant properties total phenolic content (TPC) and index (TPI), and individual polyphenols (P1–P25 represent the number of polyphenols identified in plant-based protein supplements—Table 1); I–VI—clusters.

We also determined the loading factor scatterplot and observed significant variable clustering (Figure 4). The score plot of the first two principal components (accounting for 68.65% of the total data variance) revealed the separation of the analyzed samples into different clusters in terms of the quality parameters examined. Cluster I is solely formed via the pea protein and this sample is characterized by high syringic acid, quercetin-3-*O*-galactoside, and quercetin-3-*O*-glucoside. The rice protein and ferulic acid formed cluster II. Also, cluster III consists of two variables (pumpkin seed protein and gentisic acid). Hemp protein with gallic acid, *p*-coumaric acid, salicylic acid, and rutin created cluster IV. Cluster V is solely formed via the soy protein, ellagic acid, isorhamnetin-3-*O*-glucoside, and (+)-catechin. Last, cluster VI is created via the antioxidant assays (ABTS and DPPH), TPC, and five polyphenols (kaempferol-3-*O*-rutinoside, kaempferol, *m*-coumaric acid, isorhamnetin-3-*O*-rutinoside, and myricetin).

**Figure 5:**
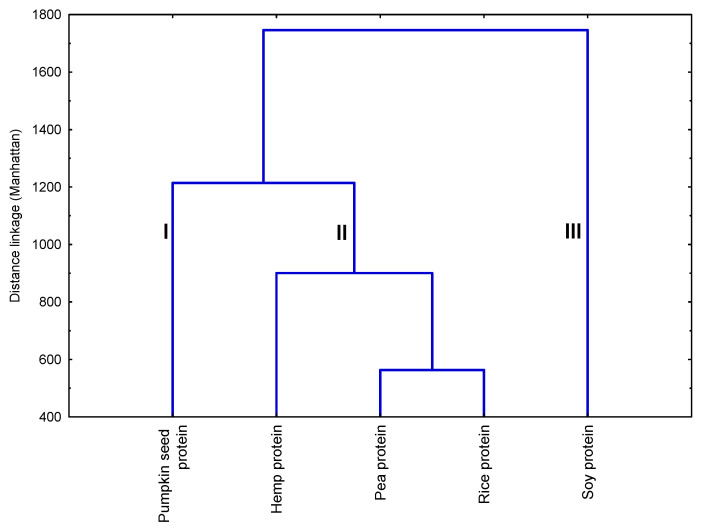
A dendrogram was obtained via cluster analysis based on Ward’s Linkage using Manhattan Distances on the individual polyphenols content in analyzed plant-based protein supplements. I–III—numbers of clusters.

In addition to the PCA and better detection of relative similarity of difference between plant-based protein supplements, cluster analysis (dendrogram) was applied to a matrix linking individual phenolic content to samples of proteins (Figure 5). The general structure of the dendrogram showed the existence of three main clusters. The first cluster included pumpkin seed protein, characterized by the highest content of gentistic and salicylic acids. The second cluster contained hemp, pea, and rice proteins, which comprised one subgroup represented by pea and rice proteins. The soy protein created a third cluster, which was the most different from clusters I and II. The dissimilarity measures soy protein may be the highest number of phenolic compounds identified and the highest concentration of ellagic acid and (+)-catechin determined in this sample. Moreover, isorhamnetin-3-*O*-rutinoside was found only in soy protein samples. The results indicate distinct differences in quality parameters across the analyzed samples. This highlights the unique characteristics of each protein source and their associated polyphenolic compounds, offering valuable insights into their relationships.

## 3. Materials and Methods

### 3.1. Chemicals

Reagents of Mass Spectrometry grade, including acetonitrile, methanol, water, and formic acid, were purchased from Sigma Chemical Co. (St. Louis, MO, USA). Also, Folin–Ciocalteu’s phenol reagent was obtained from Sigma Chemical Co. Analyzed phenols included protocatechuic acid, m-hydroxybenzoic acid, chlorogenic acid, salicylic acid, caffeic acid, syringic acid, sinapic acid, ferulic acid, *p*-coumaric acid, m-coumaric acid, o-coumaric acid, gallic acid, o-hydroxybenzoic acid, 3,4-dihydroxyphenylacetic acid, trans-cinnamic acid, vanillic acid, benzoic acid, ellagic acid, vitexin, rutin, (+)-catechin, apigenin, kaempferol, orientin, naringenin, myricetin, isorhamnetin-3-*O*-rutinoside, isorhamnetin-3-*O*-glucoside, kaempferol-3-*O*-rutinoside, quercetin, and its derivativeswere purchased from Sigma Chemical Co. (St. Louis, MO, USA). Folin–Ciocalteu’s phenol reagent, 2,2′-azinobis(3-ethylbenzothiazoline-6-sulfonic acid) diammonium salt (ABTS), 2,2-di(4-tert-octylphenyl)-1-picrylhydrazyl (DPPH), and 6-hydroxy-2,5,7,8-tetramethylchroman-2-carboxylic acid (Trolox) were obtained from Sigma Chemical Co. (St. Louis, MO, USA).

### 3.2. Research Material

Plant-based protein supplements, including soy (85% protein), rice (83.5% protein), pea (83.4% protein), hemp (49% protein), and pumpkin seed (65% protein) proteins, were purchased from a sports nutrition and supplements store in Olsztyn, Poland. The obtained samples were stored away from light at room temperature until the analysis.

### 3.3. Extraction Procedure

The extraction of phenols from plant-based protein supplements was carried out using a mixture of water/methanol (20/80, *v*/*v*). A sample (300 mg) was extracted by 30 s vortexed with 1 mL of the above solvent. Next, the mixture was sonicated for 30 s (VC 750, Sonics & Materials, Newtown, CT, USA), vortexed, sonicated, and centrifuged (Micro star 30R, VWR, Radnor, PA, USA) for 10 min (14,000 RPM, 4 °C). The obtained supernatant was collected in a 5 mL flask. The procedure was repeated five times with 1.0 mL of residual solvent. The analysis was carried out in triplicate. The obtained extracts were stored at −24 °C until further analysis.

### 3.4. Phenols Analysis

The chromatographic analysis of phenols was performed according to the methodology described by Sawicki et al. [50]. Qualitative and quantitative phenol analyses were carried out using a UHPLC system (Nexera XR, Shimadzu, Japan) coupled with a diode area detector (DAD) and a mass spectrometer (LCMS-2020, Shimadzu, Japan). Measurement parameters were as follows: eluent 0.01% formic acid in water with 2 mM of ammonium formate (A) and 0.01% formic acid in 95% acetonitrile solution with 2 mM of ammonium formate (B); flow rate of 0.15 mL/min; scanning in negative ionization; column C18 BEH (1.7 μm particle size; 100 × 2.1 mm; Waters, Warsaw, Poland); oven temperature was 50 °C; sample injection volume of 10 µL. An analysis was conducted in the selected ion monitoring mode (SIM). The analyzed compounds were identified based on their qualitative ions, retention times, and λmax value with the previously published data [50,51]. The quantity of phenols was calculated from the UHPLC-DAD-MS peak area against commercially available standards. The phenolic compound concentrations of the solutions ranged from 0.01 to 150 μg/mL, with correlation coefficients of 0.997–0.999.

### 3.5. Total Phenolic Content (TPC) Analysis

The total phenolic content (TPC) was obtained using Folin’s phenol reagent according to the procedure described by Horszwald and Andlauer [52]. A mixture containing 15 μL of appropriately diluted extract fractions and 240 μL of Folin’s phenol reagent was placed into the wells of microplates and incubated for 10 min at room temperature (RT). Next, 15 μL of 20% sodium carbonate was added and shaken. Absorbance was measured at 765 nm using a microplate reader (FLUOstar Omega, BMG LABTECH, Ortenberg, Germany). The results were expressed as mg of gallic acid (GAE)/g of sample. The linearity range of the calibration curve was from 0.062 to 0.50 mg/mL (R^2^ = 0.999).

### 3.6. Antioxidant Capacity

The ABTS and DPPH assays described by Horszwald and Andlauer [52] were used to evaluate the antioxidant capacity of the obtained plant-based protein extracts. The absorbance was measured at 734 nm (ABTS assay) and 517 nm (DPPH assay) using a microplate reader (FLUOstar Omega, BMG LABTECH). Results were presented as µmol Trolox equivalents (TE) per gram of sample. All measurements were performed in triplicate.

### 3.7. Statistical Analysis

The data distribution was evaluated using a Shapiro–Wilk test and presented as mean values and standard deviation of triplicate measurement. The differences in mean values between samples were tested using one-way analysis of variance (ANOVA) with Tukey’s HDS post hoc test or Student’s *t*-test when individual phenolic compounds were identified only in two types of plant-based protein supplements. Correlation analysis was performed using Pearson’s correlation coefficient test and presented on a heat map chart. The strength of correlation was considered fair (<0.3), moderate (0.3 to <0.5), good (0.5 to <0.7), or very good (≥0.7). Principal Component Analysis (PCA) was carried out to identify clusters of plant-based protein supplements and examine their parameters. The level of statistical significance was defined in all analyses at a *p*-value ≤ 0.05. To detect the relative similarity of the difference in individual polyphenol content between plant-based protein supplements, a cluster analysis based on Ward’s linkage method using Manhattan Distances was applied. Statistical analysis was performed with TIBCO^®^ Statistica™ ver. 13.3 (TIBCO Software Inc., Tulsa, OK, USA).

## 4. Conclusions

In conclusion, this study fills a gap in the literature by providing the first comprehensive analysis of phenolic compounds in commercially available plant-based protein supplements. Our findings reveal diverse phenols across soy, rice, pea, hemp, and pumpkin seed protein supplements, shedding light on their nutritional value beyond their protein content. We identified twenty-five phenols with predominant phenolic acids, followed by flavonoids and ellagitannins. This comprehensive analysis of phenolic content in plant-based protein supplements has significant implications for dietary recommendations and food policy. Given the growing interest in plant-based diets for health and environmental reasons, the results obtained in the current study may inform nutritional guidelines and encourage the inclusion of diverse plant proteins in diets. Moreover, recognizing the specific health benefits associated with the phenolic profiles of these supplements may aid in developing targeted dietary strategies to combat chronic diseases.

## Figures and Tables

**Table 1:** The profile and content of phenols (μg/g sample) detected in the plant-based protein supplements.

No.	Identified Phenolics	Plant-Based Protein Supplements				
		Soy Protein	Rice Protein	Pea Protein	Hemp Protein	Pumpkin Seed Protein
		phenolic acids				
P1	gallic acid	21.63 ± 0.18 ^c^	27.48 ± 0.10 ^b^	21.57 ± 0.13 ^c^	44.45 ± 1.30 ^a^	22.05 ± 0.22 ^c^
P2	*p*-coumaric acid	10.90 ± 0.11 ^b^	9.97 ± 0.01 ^c^	11.12 ± 0.10 ^b^	12.13 ± 0.07 ^a^	11.68 ± 0.12 ^a^
P3	gentisic acid	46.17 ± 1.47 ^b^	5.04 ± 0.05 ^c^	12.90 ± 0.43 ^c^	35.44 ± 0.90 ^b^	426.53 ± 6.70 ^a^
P4	caffeic acid	nd	29.64 ± 0.04 ^b^	nd	31.79 ± 0.08 ^a^	nd
P5	syringic acid	nd	nd	81.24 ± 5.24	nd	nd
P6	vanillic acid	10.71 ± 0.68 ^b^	34.33 ± 0.20 ^a^	33.35 ± 2.22 ^a^	28.27 ± 0.84 ^a^	31.91 ± 1.75 ^a^
P7	benzoic acid	33.55 ± 2.47 ^d^	811.42 ± 10.71 ^a^	725.66 ± 15.23 ^b^	776.71 ± 7.58 ^a^	605.15 ± 10.69 ^c^
P8	*m*-coumaric acid	28.98 ± 0.68 ^a^	nd	nd	25.45 ± 0.03 ^b^	30.33 ± 0.07 ^a^
P9	salicylic acid	6.86 ± 0.37 ^d^	15.20 ± 0.48 ^c^	13.29 ± 0.36 ^c^	144.35 ± 0.63 ^b^	162.82 ± 0.08 ^a^
P10	ferulic acid	19.74 ± 0.39 ^b^	36.43 ± 0.33 ^a^	17.39 ± 0.13 ^c^	19.06 ± 0.02 ^b^	18.97 ± 0.09 ^b^
P11	*o*-coumaric acid	26.65 ± 0.08 ^a^	nd	26.05 ± 0.08 ^b^	26.14 ± 0.11 ^b^	26.26 ± 0.06 ^b^
		flavonoids				
P12	(+)-catechin	67.05 ± 1.81 ^a^	nd	22.03 ± 1.15 ^b^	nd	8.94 ± 0.17 ^c^
P13	isorhamnetin-3-*O*-glucoside	22.28 ± 0.20	nd	nd	nd	nd
P14	kaempferol	20.67 ± 0.11 ^b^	nd	nd	22.84 ± 0.01 ^a^	20.70 ± 0.14 ^b^
P15	myricetin	25.31 ± 0.15 ^a^	nd	nd	22.18 ± 0.21 ^b^	nd
P16	quercetin-3-*O*-glucoside	nd	nd	23.48 ± 1.52 ^a^	nd	20.06 ± 0.06 ^a^
P17	rutin	6.49 ± 0.03 ^b^	nd	nd	166.92 ± 0.21 ^a^	nd
P18	isorhamnetin-3-*O*-rutinoside	21.88 ± 0.05 ^b^	nd	nd	23.45 ± 0.04 ^a^	nd
P19	quercetin-3-*O*-galactoside	nd	nd	53.39 ± 3.40 ^a^	nd	24.07 ± 0.24 ^b^
P20	quercetin-3-*O*-vicianoside	nd	nd	97.48 ± 3.79 ^a^	56.63 ± 0.86 ^b^	67.40 ± 0.27 ^c^
P21	kaempferol-3-*O*-rutinoside	55.70 ± 2.00 ^b^	34.22 ± 0.39 ^c^	nd	116.69 ± 1.93 ^a^	20.63 ± 0.01 ^d^
P22	quercetin	41.01 ± 0.26 ^d^	80.96 ± 1.59 ^a^	79.34 ± 1.13 ^ab^	68.21 ± 4.96 ^bc^	56.98 ± 1.37 ^c^
P23	naringenin	17.41 ± 0.08 ^b^	19.73 ± 0.09 ^a^	nd	nd	17.56 ± 0.06 ^b^
P24	apigenin	nd	3.10 ± 0.02 ^b^	4.75 ± 0.04 ^a^	nd	nd
		ellagitannins				
P25	ellagic acid	118.35 ± 7.05 ^a^	49.59 ± 0.46 ^b^	nd	nd	28.19 ± 2.16 ^c^
	TPI	601.34 ± 16.56 ^c^	1157.12 ± 11.17 ^b^	1223.02 ± 29.02 ^b^	1620.69 ± 1.57 ^a^	1600.24 ± 2.43 ^a^

The results are expressed as the means ± SD. Different letters depict statistically significant differences (*p* ≤ 0.05) in the same row. TPI—Total Phenolic Index calculated by the sum of individual phenolics identified in the tested samples.

**Table 2:** The antioxidant capacity of plant-based protein supplements.

Samples	Antioxidant Activity Assays	
	DPPH	ABTS
	µmol TE/g	µmol TE/g
soy protein	4.12 ± 0.10 ^c^	6.26 ± 0.07 ^b^
rice protein	0.84 ± 0.06 ^d^	0.58 ± 0.03 ^d^
pea protein	nd	1.95 ± 0.04 ^c^
hemp protein	9.01 ± 0.40 ^a^	9.37 ± 0.47 ^a^
pumpkin seed protein	4.93 ± 0.13 ^b^	5.68 ± 0.13 ^b^

The results are expressed as the mean ± SD. Different letters depict statistically significant differences (*p* ≤ 0.05) in the same column. nd—not detected. TE—Trolox equivalent.

## Data Availability

The raw data supporting the conclusions of this article will be made available by the authors upon request.
